# Alcohol Dependence and Altered Engagement of Brain Networks in Risky Decisions

**DOI:** 10.3389/fnhum.2016.00142

**Published:** 2016-03-31

**Authors:** Xi Zhu, Kelsey Sundby, James M. Bjork, Reza Momenan

**Affiliations:** ^1^Clinical NeuroImaging Research Core, National Institute on Alcohol Abuse and Alcoholism, National Institutes of Health, BethesdaMD, USA; ^2^Department of Psychiatry, Virginia Commonwealth University, RichmondVA, USA

**Keywords:** decision-making, risk, ICA, alcohol-dependence, salience network

## Abstract

Alcohol dependence is associated with heightened risk tolerance and altered decision-making. This raises the question as to whether alcohol dependent patients (ADP) are incapable of proper risk assessment. We investigated how healthy controls (HC) and ADP engage neural networks to cope with the increased cognitive demands of risky decisions. We collected fMRI data while 34 HC and 16 ADP played a game that included “safe” and “risky” trials. In safe trials, participants accrued money at no risk of a penalty. In risky trials, reward and risk simultaneously increased as participants were instructed to decide when to stop a reward accrual period. If the participant failed to stop before an undisclosed time, the trial would “bust” and participants would not earn the money from that trial. Independent Component Analysis was used to identify networks engaged during the anticipation and the decision execution of risky compared with safe trials. Like HC, ADP demonstrated distinct network engagement for safe and risky trials at anticipation. However, at decision execution, ADP exhibited severely reduced discrimination in network engagement between safe and risky trials. Although ADP behaviorally responded to risk they failed to appropriately modify network engagement as the decision continued, leading ADP to assume similar network engagement regardless of risk prospects. This may reflect disorganized network switching and a facile response strategy uniformly adopted by ADP across risk conditions. We propose that aberrant salience network (SN) engagement in ADP might contribute to ineffective network switching and that the role of the SN in risky decisions warrants further investigation.

## Introduction

Effective decision-making requires the integration of a variety of cognitive processes and the cooperation of multiple neural networks (Bickel et al., 2007). Even basic decision-making, for example, incorporates numerous mental processes ranging from the extraction of information from working memory to the merging of affective and rational information (Bechara and Damasio, 2005). When risk is involved and the decision requires the weighing of potential rewards and penalties, the demands on cognitive resources becomes even greater (Bechara et al., 1994; Elliott et al., 2000; Ernst et al., 2002; Gonzalez et al., 2005). The introduction of risk into the decision-making paradigm may, therefore, require a distinct set of neural networks to respond to the additional cognitive demands.

Alcohol dependence (AD) is often associated with aberrant risk-reward evaluation, particularly when a decision involves potential penalties (Bechara et al., 2001, 2002; Bechara and Martin, 2004). Specifically, several task-based neuroimaging studies have documented that substance dependent individuals disproportionately elect immediate rewards at the risk of future penalties, and a variety of brain regions have been linked to this behavior. For example, dampened activation in the left pregenual anterior cingulate cortex (ACC) was found in drug abusers compared to control subjects in a risky decision-making task (Fishbein et al., 2005). Similarly, disrupted risk-related processing in the ACC and insula were associated with methamphetamine-dependent individuals (Gowin et al., 2014). Also, substance dependent patients demonstrated blunted recruitment of the conflict-monitoring neurocircuitry of the posterior mesofrontal cortex when faced with a conflict between concurrently increasing reward and risk of penalty (Bjork et al., 2008).

Previous work has also begun to identify differences in neural networks in ADP. Although several resting-state studies have reported altered functional connectivity reflecting differences in the strength of correlation between regions in the salience network (SN), the executive control network (ECN), and the basal ganglia thalamus network (BGTN) in AD, no study to date has attempted to examine how these brain networks may be engaged differently in ADP during decisions involving risk. (Chanraud et al., 2011; Beck et al., 2012; Sullivan et al., 2013; Motzkin et al., 2014; Zhu et al., 2015a,b). To fill this important gap, the first aim of the current study is to examine how networks are engaged differently for risky compared to safe decisions. Specifically, our analysis may reveal how large-scale network engagement varies between safe and risky decisions in healthy controls (HC) and how this distinction might be lost or mitigated in ADP. By using Independent Component Analysis (ICA), the current study aims to expand on findings published by Bjork et al. (2008) and to identify differences in neural recruitment in ADP during risky decisions on a network level as compared to independent brain regions. Of interest, is whether the blunted neural activation of the ACC during the decision conflict that was reported in Bjork et al. (2008) may have resulted from adoption of a cognitively facile response strategy, with a concomitant failure to engage networks that actively processes risk and variable outcomes in trials with a potential for penalty. A network approach may provide a more comprehensive understanding of how the brain of ADP manages decisions and accounts for risk. Together these findings may give important clinical insights into the treatment of this disease.

Independent Component Analysis is an analytical tool that can be used to identify networks engaged during a task (Calhoun et al., 2001, 2009). ICA may provide a more accurate depiction of cognitive function by revealing subtleties masked in the traditional general linear model (GLM). Specifically, it allows the study to tease apart how overlapping networks are independently contributing to the overall change in hemodynamic response (Perlbarg et al., 2008). Additionally, ICA is able to detect how the same brain region may be involved in multiple networks exhibiting distinct behaviors. One network, for example, may respond to stimuli via increased engagement, while an overlapping network may be disengaged by the same stimuli, where brain regions shared between the two networks would show no activity in a traditional task-based GLM. That a region is incorporated in multiple networks being simultaneously engaged and disengaged by the same task is the type of nuanced neural behavior that may be overlooked in traditional GLM analysis as the deactivation and activation may cancel (Xu et al., 2013a,b). We applied ICA to data previously used in a GLM analysis to examine network engagement during safe and risky decision-making, how this may vary in ADP, and how multiple networks involving the same brain regions may act differently.

Disparities in how HC and ADP employ networks to handle risky decisions may be particularly apparent in networks that are important for identifying risk as a salient cue and for coordinating subsequent processing. When confronted with challenges in a task such as distractions or in the case of risky decisions, potential penalties, recruitment of regulatory, or attentional networks are used to prevent a decline in task performance despite the added challenges. Such increases in top-down control mechanisms are triggered by incentives to optimize goal directed behavior by selectively attending to important information and performing network switches to aid in further processing (Sarter et al., 2006). The SN is a neural network involved in detecting salient stimuli, allocating attention, shifting operations between large neural networks (i.e., networks involved with activities like attention and memory), and guiding behavioral responses (Menon and Uddin, 2010; Sullivan et al., 2013). If HC do in fact significantly modify network engagement when dealing with risky decisions, the SN may assume a more prominent role by facilitating the necessary switching between networks and modulating attentional processing in service of actionable stimuli, such as instrumental rewards (Sarter et al., 2006). Altered SN engagement during a risky decision, on the other hand, might impair the engagement of other networks used to aid in the decision-making process. Evidence of lower SN task driven connectivity in ADP has previously been hypothesized to cause interference with effective switching between networks and, thus, contribute to a decline in executive control (Sullivan et al., 2013). Despite evidence of altered connectivity, the current literature has yet to explore whether changes in SN connectivity translate to altered SN engagement during behaviors relevant to AD, such as risky decision-making. Efforts to model altered network connectivity in nicotine addiction have, however, proposed aberrant SN engagement as a source of disrupted network dynamics and in turn, diminished cognitive performance. The above theory specifically focuses on the insula as a region that may mediate aberrant SN recruitment in addiction (Sutherland et al., 2012).

The insula, a primary node in the SN and a region implicated in both AD and decision-making, is a complicated region to understand independently and may particularly benefit from a network approach (Menon and Uddin, 2010; Naqvi and Bechara, 2010). Three functional subdivisions of the insula have been identified based on a meta-analysis of human neuroimaging data: (1) posterior insula accounting for sensorimotor, pain, and language processing; (2) dorsal anterior insula engaged in higher executive control function; and (3) ventral anterior insula responsible for social–emotional processing and autonomic function (Droutman et al., 2015). Inconsistent and diverse findings from previous studies make it difficult to condense the insula’s function into a single hypothesis as to how altered insula activity may manifest itself in AD. For example, decreased craving in cigarette smokers with insula damage may lead to hypotheses of a hyperactive insula in dependence (Naqvi et al., 2007). Conversely, evidence associating insula activation with aversive somatic markers and risk avoidance could suggest that high risk tolerance in ADP may be related to diminished insula activity (Paulus et al., 2003). The varied functions paired with findings implicating distinct divisions of the insula in separate networks suggests that the insula may in fact be working differently in multiple networks (Taylor et al., 2009; Droutman et al., 2015). The current literature has yet to examine whether the insula plays different roles in different networks in risky decision-making tasks. The second goal of the current study is to address this question by using a network analysis approach.

The present study aimed to test three primary hypotheses: (1) First, as indicated by previous findings, we hypothesized that HC would employ additional brain utilities to account for the increased cognitive strain posed by decisions involving risk (Bjork et al., 2008). Because ADP is associated with altered decision-making and heightened risk tolerance, we hypothesized that ADP, unlike HC, would not conduct the appropriate shifts in network engagement to distinguish between risky and safe decisions. (2) Specifically, we hypothesized that HC would recognize risky decisions as salient events and exhibit increased SN engagement in risky decisions compared to safe decisions. Conversely, we predicted abnormal SN engagement in ADP during risky decisions, which in turn may partially account for our first hypothesis. (3) Lastly, with the insula being an important hub in the SN, we expected ICA to reveal multiple functional networks involving different parts of the insula and that these networks may exhibit distinct behaviors during decision-making (Menon and Uddin, 2010).

## Materials and Methods

### Participants

Sixteen alcohol dependent patients (ADP; seven females) with an average age of 32.9 years (range: 18–43, std: 7.2) and 34 healthy controls subjects (HC; 18 females) with a mean age at examination of 31.9 years (range: 23–46, std: 5.7) participated in the study (see **Table 1**). Age was not different between the two groups (*t* = 3.3, *p* = 0.65). The majority of subjects were originally analyzed in Bjork et al. (2008), with the inclusion of 17 additional controls in the present study. All participants were right-handed and free of neurological disease and other significant histories of illness as determined by Structured Clinical Interviews for DSM-IV. Controls were recruited through community advertisements and information notices distributed in the Washington, DC, USA metropolitan area. ADP were recruited from the inpatient alcohol treatment unit at the National Institutes of Health Clinical Research Center in Bethesda, Maryland. Patients with a history of seizures, IQ < 80, psychosis, or craniofacial features indicative of fetal alcohol syndrome were excluded. All ADP met DSM-IV criteria for AD. Study procedures were approved by the Institutional Review Board of the National Institute on Alcohol Abuse and Alcoholism and written informed consent for the study was obtained from all of the subjects. Participants were compensated for their time.

**Table 1:** Demographic and clinical profile of the participants in this study.

	ADP	HC
*N*	16	34
Gender M (F)	9 (7)	16 (18)
Smoker	15	6
Age (years)	32.9 (7.2)	31.9 (5.7)
Ethnicity (AA/C/O)	11/05/00	07/22/04
Years of heavy drinking	10.5 (6.5)	N/A

*AA, Africa American; C, Caucasian; O, others.*

### Task Design

The risk-taking task (RTT) used in the present study has been described in Bjork et al. (2008). Briefly, the RTT presented subjects with four types of pseudorandomly presented trials (duration 14 s, *n* = 24 each) that varied by the level of risk of a penalty. The screen displayed a cumulative winnings counter for the duration of the entire trial and distinct screen colors were used to inform subjects of the trial type. Subjects were required to press a button twice during each trial, once to initiate the trial and once when either voluntarily opting to end the trial themselves or responding to a cue indicating the end of the trial. The RTT includes motor control trials (MC), no-penalty (NP) trials with a guaranteed reward, low-penalty trials (LP), and high-penalty trials (HP), (see Supplementary Materials).

In NP trials with a guaranteed reward, indicated by a green background, subjects began accruing money after a first button press in response to a “$” cue, and accumulated winnings throughout the full duration of the trial with no chance of penalty. After 4, 6, 8, or 10 s into the trial, subjects were prompted to press the button a second time when the word “press” appeared on the screen signifying the end of the trial. In LP trials, with a yellow background, subjects again began accruing money after the first press in response to the $ cue. Each LP trial had an undisclosed time limit (4, 6, 8, or 10 s) for money accrual. If the subject voluntarily stopped reward accrual before the secret time limit by pressing the button (top bifurcated outcome), he or she added the accrued winnings from that trial to total winnings. If the participant failed to stop reward accrual before the secret time limit, he or she “busted” and forfeited all winnings from that trial, and was instructed to press the button a second time to ensure uniform motor behavior. In HP trials, indicated by a red background, the trial had the same design as LP trials except that a “bust” resulted in the subtraction of money from previous earnings. The current study aimed to investigate risky decisions that present a cognitive conflict requiring individuals to weigh the risk of penalty compared to the value of a potential reward. Given the high stakes, it may be an obvious decision to avoid risk in the HP trials for both ADP and HC. Indeed, subjects uniformly took few risks in the HP condition. Therefore, because the LP trials may be a more conflicting condition that requires more processing and neural recruitment to determine if the risk is worth a potentially higher reward, we focused our analysis on the other two incentivized task conditions, NP and LP.

### Image Acquisition and Analyses

Images were collected using a 3T General Electric MRI scanner (General Electric, Milwaukee, WI, USA). We acquired twenty-four 3.8 mm-thick axial slices with a 1 mm gap sequentially from inferior to superior. We used a T2^∗^-sensitive echoplanar sequence with a repetition time (TR) = 2000 ms, echo time (TE) = 40 ms, flip angle = 90°, and in plane resolution = 3.75 mm × 3.75 mm. We conducted structural scans with a T1-weighted MP-RAGE sequence (TR = 100 ms; TE = 7 ms; flip angle = 90°) for co-registration. A head cushion was used to limit head motion.

Image preprocessing was performed using AFNI including (1) slice acquisition timing alignment, (2) 3D motion-correction, (3) spatial smoothing (rms = 4 mm), (4) voxel-wise despiking of signal and bandpass filtering. The detailed information was presented in Bjork et al. (2008).

Our analysis featured two key steps: (1) identify the functional networks engaged during performance of the RTT using group ICA applied to all subjects, and (2) identify individual subjects’ spatial maps and associated timecourses corresponding to the group ICA maps using the Group ICA of fMRI Toolbox (GIFT) program (http://icatb.sourceforge.net/) as a MATLAB toolbox (Matlab6R2013b, Mathworks Inc., Sherborn, MA, USA). All functional runs from all 50 subjects were included in a single Group ICA analysis. Two data reduction steps were used through principal component analysis (PCA). Data were first reduced for each subject’s functional data (Calhoun et al., 2001; Erhardt et al., 2011). The subjects were then concatenated into one group and put through another data reduction step. Independent components, or networks, were calculated using the Infomax algorithm (Bell and Sejnowski, 1995). We identified 20 components with ICA, which characterized 85% of the variance. Single-subject component timecourses were then back reconstructed and each subject’s component image was converted to a *z*-score image to facilitate between-subjects comparisons.

Statistics were performed on the group of subjects for each of the networks identified by ICA. To identify those components most involved in each trial type, a GLM analysis was used. We examined the role of each component at trial anticipation (onset of trial) and at the decision execution (time of second button press) and how this differed according to trial type. Anticipatory activation was time-locked to the onset of a trial, when the screen color signaling the trial type first appeared. The decision execution was operationalized as second motor responses: terminating reward in successful LP trials; second button presses prompted by a cue in NP. These two events were modeled using the canonical hemodynamic response function (HRF) in SPM12b to examine the association between component timecourses and different trial types (Welcome Functional Imaging Laboratory, London, UK). The resulting β-weights, a measure of each component’s temporal relation to trial-types, were then entered into paired *t*-tests to identify those components significantly more engaged during LP or NP trials at both anticipation and decision execution. A FWE-corrected significance threshold of *p* < 0.05 was used.

## Results

### Behavior

Addition of control subjects did not change the similar risk-taking behavioral outcome between ADP and HC reported in Bjork et al. (2008). There was no difference in the number of busts between ADP and HC in LP trials. We also found no significant differences in the money earned by HC and ADP during the task. Similarly, there was no significant difference between the amount of time between first and second presses for ADP and HC (see Supplementary Table S5).

### Network Identification

The ICA decomposition resulted in 20 spatial component maps, which revealed all classically identified resting state networks, including default mode network (DMN), executive control network (ECN), visual network, somatosensory network, auditory network, and artifactual networks (see Supplementary Figure S1). Brain stem, CSF, and head motion were identified as artifactual networks and removed from the study.

#### Network Engagement in No-Penalty and Low-Penalty Trials

##### Network engagement during decision anticipation

To examine whether HC and ADP engaged networks differently during the anticipation of risky compared to safe decisions, we conducted within group comparisons of network engagement between safe (NP) and risky trials (LP). The results of the within group comparisons at anticipation are summarized in **Table 2**. Any networks not well established in the literature are labeled according to a prominent node in the network and key structures in the network have been provided for more information.

**Table 2:** Network engagement distinguishing safe and risky trials at anticipation.

		HC		ADP	
		LP vs. NP		LP vs. NP	
**Network**	**Structures**	**FWE-*p***	***t***	**FWE-*p***	***t***

SN	Dorsal anterior insula, OFC, ACC	0.010^∗^	3.653	0.000^∗∗^	6.038
ACC1	ACC	0.293	0.780	0.000^∗∗^	4.609
Posterior Insula	Posterior insula	0.119	-1.483	0.000^∗∗^	-6.381
ECN1	FP, mPFC	0.045^∗^	2.459	0.000^∗∗^	4.202
ECN2	AG, postcentral gyrus	0.053	-2.016	0.000^∗∗^	-4.333
Motor 1	Precuneus	0.053	2.145	0.000^∗∗^	4.238
RECN	IFG, OFC	0.053	2.091	0.000^∗∗^	4.235
Motor 2	ACC, precentral gyrus	0.020^∗^	3.042	0.000^∗∗^	4.056
DMN	PCC, mPFC	0.045^∗^	-2.443	0.011^∗^	-2.569
BGTN	Thalamus, accumbens, caudate	0.053	2.033	0.000^∗∗^	4.473

*A positive *t*-value indicates higher engagement in the first trial type listed in the comparison. Networks with significantly different levels of engagement between trial types. ^∗^*p* < 0.05, ^∗∗^*p* < 0.001.*

Our findings indicate that both groups engaged networks differently during the anticipation of risky (LP) compared with safe decisions (NP). Both groups demonstrated an overall increase in network engagement when anticipating a decision involving risk. A subset of networks, however, revealed higher engagement during anticipation of safe decisions. Specifically, both groups engaged the SN, Motor 2 network, and ECN1 more when anticipating LP versus NP trials. ADP additionally demonstrated greater engagement of the ACC1, Motor1, RECN, and BGTN networks at anticipation of LP. Conversely, both HC and ADP exhibited higher engagement of the DMN at anticipation of NP versus LP trials. Only ADP showed higher engagement of the posterior insula network and ECN2 when anticipating NP trials.

##### Network engagement during decision execution

We also compared network engagement between safe and risky trials at the decision execution. The results of the within group comparisons at decision execution are summarized in **Table 3**.

**Table 3:** Network engagement distinguishing safe and risky trials at decision execution.

		HC		ADP	
		LP vs. NP		LP vs. NP	
**Network**	**Structures**	**FWE-*p***	***t***	**FWE-*p***	***t***

SN	Dorsal anterior insula, OFC, ACC	0.013^∗^	2.863	0.107	1.982
ACC1	ACC	0.015^∗^	2.685	0.107	1.885
Posterior insula	Posterior insula	0.043^∗^	-2.181	0.608	-0.347
ECN1	FP, mPFC	0.000^∗∗^	4.645	0.170	1.573
ECN2	AG, postcentral gyrus	0.000^∗∗^	-6.269	0.010^∗^	-3.475
RECN	IFG, OFC	0.015^∗^	2.647	0.107	1.877
BGTN	Thalamus, accumbens, caudate	0.003^∗^	3.478	0.608	0.662

*A positive *t*-value indicates higher engagement in the first trial type listed in the comparison. Networks with significantly different levels of engagement between trial types. ^∗^*p* < 0.05, ^∗∗^*p* < 0.001.*

We found that HC continued to exhibit significantly different network recruitment for risky and safe trials at decision execution. HC engaged the SN, ECN1, RECN, BGTN, and ACC1 networks more in LP compared with NP trials at decision execution. Contrariwise, for NP trials HC showed higher engagement of ECN2 and the posterior insula network at the decision execution. ADP only significantly varied network engagement in one network at decision execution, exhibiting increased engagement of ECN2 in NP trials.

#### Specific Networks of Interest

##### Network recruitment in insula

**FIGURE 1:**
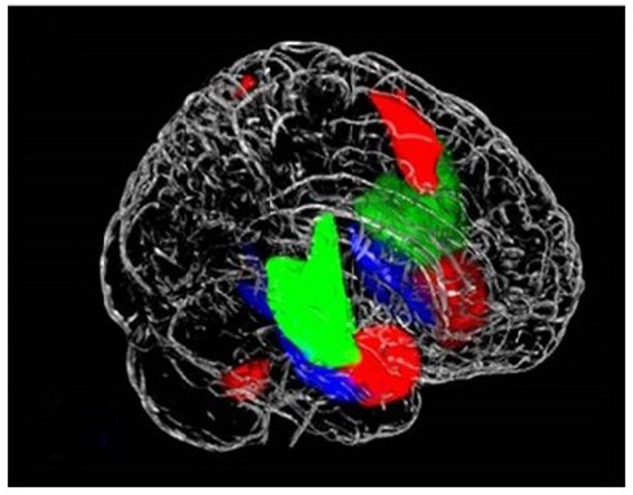
**Three networks that contain the insula, (1) posterior insula (green) accounts for sensorimotor, pain, and language processing; (2) dorsal anterior insula/salience network (red) engaged in higher executive control functions; and (3) ventral anterior insula (blue) responsible for social–emotional processing and autonomic function**.

**FIGURE 2:**
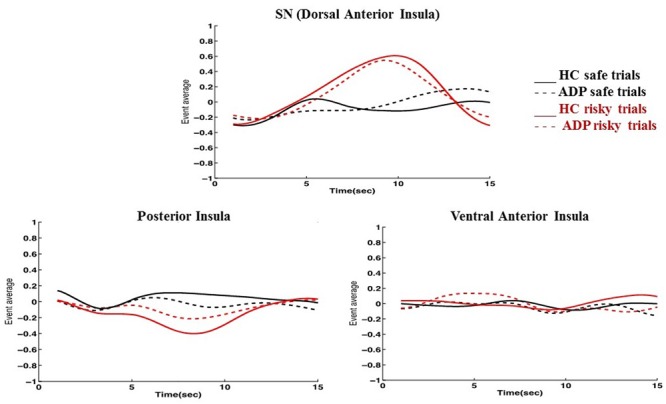
**Graphs depicting how the insula networks are engaged for each trial type across the duration of a trial indicate several key findings.** (1) The SN/dorsal anterior insula is engaged more in risky trials (LP) than safe trials (NP) by both HC and ADP; (2) The posterior insula network exhibits the inverse behavior and is more engaged for safe trials (NP) than risky trials (LP) by both HC and ADP; (3) ADP show a dampened but similar pattern of SN engagement in risky trials compared to HC during anticipation; (4) ADP show over-engagement of the posterior insula network compared to HC during risky trials (LP) at anticipation (5) The ventral anterior insula network does not show significant changes in engagement during any of the trial conditions.

Consistent with previous reports on the functional parcellation of the insula, ICA identified three distinct networks engaged during the task that involved the insula: the SN encompassing the dorsal anterior insula, a network involving the ventral anterior insula, and a network including the posterior insula (**Figure 1**) (Droutman et al., 2015). We found increased engagement of the SN in risky compared to safe trials. Conversely, our results indicated higher engagement of the posterior insula network during safe versus risky trials. There were no differences between the task conditions in the engagement of the ventral anterior insula network (**Figure 2**).

Because of our specific interest in the insula, we also conducted between group comparisons to directly examine any potential differences in how ADP and HC engaged each of the three insula networks during the task. We found that at anticipation HC engaged the SN more than ADP for risky trials (LP). Conversely, at anticipation ADP engaged the posterior insula network more than HC for risky trials (LP). There were no significant between group differences at the decision execution. The results of the between group comparisons of the insula networks are summarized in **Table 4**.

**Table 4:** Engagement of insula networks between ADP and HC at anticipation.

	NP		LP	
Network	FWE-*p*	*t*	FWE-*p*	*t*
SN	0.261	-1.744	0.041^∗^	-2.309
Posterior insula	0.663	-0.438	0.041^∗^	2.282
Ventral anterior insula	0.663	0.462	0.163	1.418

*A positive *t*-value indicates higher engagement in ADP. Networks with significantly different levels of engagement between ADP and HC. ^∗^*p* < 0.05.*

## Discussion

In this study, we examined how ADP and HC engaged neural networks differently for risky versus safe decisions during performance of a RTT. Consistent with our first hypothesis, HC demonstrated a clear distinction in network engagement for safe compared with risky decisions. Furthermore, as predicted, ADP demonstrated a failure to appropriately shift engagement during the decision-making process to distinguish between safe and risky decisions. Corroborating our second hypothesis, HC exhibited increased engagement of the SN for risky decisions. Additionally, we found evidence of aberrant SN engagement in ADP, a network that we propose may play a role in conducting network shifts to appropriately distinguish between types of decisions.

Finally, in support of our third hypothesis, our results revealed multiple networks encompassing the insula that exhibited distinct behaviors during the task. Specifically, we identified three networks involving subdivisions of the insula: the dorsal anterior insula (part of the SN), the ventral anterior insula, and the posterior insula. Additionally, compared to HC, ADP exhibited abnormal engagement of insula networks during risky decisions. These results indicate that the insula may assume a variety of roles in the decision-making process via multiple networks, and specifically, that insula networks may be associated with altered decision-making in ADP. Together, these findings highlight the importance of a network approach to understanding complex cognitive tasks and how they may go awry.

### Behavioral Outcomes

We compared task behavior between ADP and HC to confirm that the original behavioral findings (of no systematic confounding group differences in the experience of aversive “busts”) were maintained after inclusion of additional control subjects. We found no significant differences between ADP and HC in the number of busted trials or time between first and second press, a measure of risk tolerance. We propose multiple explanations for why our findings did not reveal differences in task behavior.

First, an explanation proposed by several studies, is that higher neuroticism associated with ADP may in fact make these patients more risk averse toward monetary (as opposed to alcohol) reward. As a result, ADP may be motivated to relieve the anxiety of risk that would accrue as the trial proceeded (Bjork et al., 2008; Gilman et al., 2015). Indeed, by giving the participant the option to end the trial, our task design allows for complete risk aversion and an easy way out. Second, as suggested by Bjork et al. (2008), risk aversive behavior and avoiding busts may be appealing to ADP because it is less cognitively demanding. Consistent with this theory, reduced engagement of risk-sensitive networks as risky trials proceed may be interpreted as a signature of a deliberate uniform strategy to avoid risk to preserve certain gains, and by extension, to avoid the cognitive demands of trial-by-trial risk-taking. In contrast, HC may have been more actively engaged in trial-by-trial evaluative processes, with concomitantly increased processing of outcome uncertainty, in service of the same general behavioral outcome.

Finally, it may be that alterations in network engagement do in fact translate to riskier behavior in scenarios more relevant to substance dependence. For example, if playing the game for alcohol instead of money, our results may have revealed behavioral changes. Previous findings have identified differences in how ADP respond to various types of reward cues (i.e., alcohol vs. money, etc.), suggesting that the type of reward offered may influence study results. The above study also found evidence of altered neural activity in ADP during the expectation of a monetary reward that did not reveal any behavioral differences in the task (Wrase et al., 2007). This indicates that differences detected in imaging data may not always result in changes in task behavior. At a minimum, our results reveal important differences in the neural networks engaged during risk-reward assessments in ADP.

### Large Scale Shifts in Network Engagement for Decisions under Risky vs. Safe Conditions

To address our first hypothesis and examine whether HC engaged networks differently for risky and safe decisions and whether similar distinctions were observed in ADP, we compared networks involved in LP (“decisions under risk”) with those engaged in NP (“decisions under no risk”). We predicted that HC would recruit a larger and more diverse set of networks in response to risky conditions. Our findings indicate that both groups engaged networks differently during anticipation of risky compared with safe decisions. Specifically, both groups demonstrated an overall increase in network engagement when anticipating a decision involving risk. A subset of networks, however, revealed higher engagement during anticipation of safe decisions. These networks may be sensitive to the expected value of the trial, where safe trials typically earned over twice as much (>$1) as a risky trial with a typical voluntary stop time (40–50 c; Bjork et al., 2008). Therefore, individuals do not simply employ more networks to accommodate risk, but are discriminative in the networks engaged. This reflects literature showing that the specific brain regions employed during a decision depend on the decision conditions (i.e., risky, guaranteed, or ambiguous; Hsu et al., 2005; Krain et al., 2006). These findings suggest that like HC, ADP are capable of recognizing risk and the need to modify network engagement.

However, although ADP demonstrated distinct network engagement at anticipation of safe versus risky decisions, the set of networks engaged in ADP was different than the set engaged by HC for both trial types. For example, among other networks, ADP showed higher engagement of the ACC1 network in anticipation of risky trials while HC did not. ACC1 is a network of particular interest as it encompasses the ACC and connections to the dorsal–ventral affective components, regions associated with managing decision conflict. The original GLM findings indicated lower activation of the ACC in ADP (Bjork et al., 2008). Although ADP initially showed higher engagement of the ACC1 network at the anticipation of risky trials, they also engaged this network differently than HC at decision execution. Our results suggest that ADP may fail to continue updating conflict monitoring as they approach decision execution. In contrast to ADP, HC augment their ACC1 engagement as risk increases demonstrated by higher engagement of ACC1 at the decision execution. This network’s role in monitoring risk-reward conflict is most critical after anticipation, as this is when the conflict really occurs, as the decision gets closer to the time of potential bust. Therefore, consistent with the original GLM analysis, ADP exhibited ineffective recruitment of this network when it would perhaps be most critical for conflict monitoring (Bjork et al., 2008). In addition to recruiting networks differently, ADP also exhibited engagement of a larger number of networks during anticipation compared to HC. This may be an example of neural compensation, a mechanism through which ADP have been shown to increase neural activity of brain regions to accomplish the same behavior as HC and compensate for dampened activation in regions typically recruited for the task (Sullivan and Pfefferbaum, 2014). We suggest that in response to lower activation of some regions, such as those in the SN, ADP may require the recruitment of more networks to process the difference between safe and risky trials at anticipation. Overall, these findings demonstrate that ADP engaged networks differently than HC for both safe and risky conditions and that aberrant network engagement occurred as early as anticipation.

The critical difference between HC and ADP, however, emerged later in the decision-making process, at the decision execution. While HC showed a robust distinction in network engagement between risky and safe decisions at both trial anticipation and decision execution, ADP only made this distinction (with the exception of one network) at anticipation. Other studies have similarly revealed that while neural activation increases in HC in response to cues before the selection of a risky versus a safe alternative, the neural responses in ADP do not differ (Gilman et al., 2015). Our findings suggest that despite initially distinguishing between risky and safe conditions, ADP failed to appropriately adapt network recruitment and maintain the distinction as the decision-making process continued. This may have arisen from a preferential processing of the expected value of a trial upon initial detection of the trial type at its onset in ADP, in light of adoption of a uniform strategy. Therefore, while ADP may initially process the difference between the decision conditions, how they manage this information may vary from HC. We suggest that ADP may discount the difference and treat both conditions the same, opting for an immediate easy reward that avoids the continual processing and network recruitment that would be required if the trial were to continue. Conversely, HC may employ a more fluid continuous on-line processing of trial conditions (more flexible strategy). Unfortunately, we did not collect debriefing metrics on task strategy.

Additionally, because HC continued to update their network recruitment during safe and risky trials throughout the decision-making process, we could compare the networks engaged by HC during anticipation versus those engaged at decision execution. Our results indicate that different networks are engaged at anticipation compared with those closer to the instance of decision execution for both risky and safe trials. Consistent with previous studies showing that specific brain regions contribute differently at various points of reward and loss processing, our results suggest that the decision-making process in HC is highly dynamic and involves network shifts reflective of both the conditions of the decision and the point in the decision-making process (Rogers et al., 2004).

### The Salience Network

As predicted by our second hypothesis, we found higher engagement of the SN during risky trials in HC at both the anticipation and at the decision execution stages. Additionally, we found several instances of altered SN engagement in ADP. First, although ADP engaged the SN at anticipation of a risky decision, they failed to maintain increased engagement throughout the decision, exhibiting no difference in SN engagement between safe and risky trials at decision execution. Second, ADP demonstrated less engagement of the SN compared to HC when anticipating risky trials. These findings, indicating altered SN engagement in ADP, are consistent with studies that link impaired SN function with substance use disorders (Sutherland et al., 2012).

We believe that network switches are required to engage networks differently for risky and safe decisions and that the robust distinction in network engagement between safe and risky conditions in HC at decision execution indicates that they conducted multiple network switches to adapt to the decision type. Furthermore, because risky decisions are more cognitively demanding we expect them to involve more networks and, therefore, more network switching. As mentioned, our results did in fact reveal a larger number of networks with increased engagement during risky compared with safe trials for HC at both anticipation and at the decision execution. If the SN is involved in conducting network switches to adapt to the increased demand of risky trials, we would also predict higher SN engagement during risky trials. This was also supported in our data as we found higher SN engagement during risky trials in HC at both anticipation and decision execution. Although our study design does not allow us to make causal links between SN engagement and how other networks are engaged, our data reveals an association between higher SN engagement, an increased number of networks engaged, and risky trials. ADP also engaged a greater number of networks and demonstrated higher engagement of the SN in risky trials at anticipation. However, at the decision-execution, ADP exhibited an acute lack of distinction between network engagement in safe and risky trials, and the difference in SN engagement between trial types vanished. As proposed in Sullivan et al. (2013), this data suggests that a compromised SN in AD might contribute to impaired cognitive shifts. Specifically, we propose that aberrant SN engagement may interfere with effective network switching to adjust network engagement for risky decisions. Because our data does not provide evidence of a causal relationship between higher SN engagement and effective network switching in risky decisions, future work is needed to examine whether the recruitment of other networks during risky decisions is indeed dependent on normal SN function during risky decisions.

### The Insula Networks

Including the SN, we found three functional networks incorporating parts of the insula that were engaged differently in the task. Consistent with previously identified functional subdivisions of the insula, our results included the dorsal anterior insula (SN), ventral anterior insula, and the posterior insula in three independent networks (Droutman et al., 2015). By examining how HC engaged each of these networks during the task, we can identify the decision conditions (risky or safe) during which each of these networks is primarily engaged. As discussed, the SN (including the dorsal anterior insula) demonstrated higher engagement in risky versus safe trials. This is consistent with past work that has similarly observed engagement of networks involving the anterior insula in response to risk. In conjunction with its executive role in the SN, the engagement of the anterior insula is also related to aversive feelings and risky decisions. Specifically, the anterior insula has been implicated in adapting decision strategies and selecting safe options in financial risks (Kuhnen and Knutson, 2005). Therefore, it may be that as part of the SN, the anterior insula has a multifaceted role in risky decision-making: perceiving aversive stimuli or indications of risk and alerting the SN, and governing network shifts to cope with the added demands. In contrast to the SN, the posterior insula network was more engaged in safe trials. While the posterior insula is primarily attributed to somatic experiences, activation of the posterior insula has been linked to delaying gratification, indicating that it may also have a role in reward evaluation (Wittmann et al., 2007; Naqvi and Bechara, 2009). This finding may also reflect visceral signatures of excitement at the larger expected value of the safe trials. The ventral anterior insula network did not increase engagement for either safe or risky decisions. Together these findings suggest that the insula is involved in both safe and risky decisions but primarily contributes to each of these via distinct functional networks.

To better understand how the SN and the posterior insula network are involved specifically during risky decisions, we examined their engagement during risky trials. Although the posterior insula network is more engaged in safe versus risky trials, it may still exhibit engagement during risk but at a lower level. However, our findings indicate that the SN and posterior insula network show inverse network engagement during risky decisions. While the SN increases engagement during risky trials the posterior insula network conversely decreases engagement. The identification of multiple networks employing the insula in diverse ways under the same conditions is further supported by studies that reveal overlapping functional networks that contribute to the same study tasks in unique ways (Xu et al., 2013b). These findings reiterate the importance of a network approach in uncovering the range of behaviors exhibited by networks overlapping on the same brain regions.

Finally, we did a between group comparison to examine how the SN and posterior insula network are engaged differently by ADP and HC during risky trials. Although the posterior insula network demonstrated disengagement in both HC and ADP, ADP did not disengage the network as robustly. We found that at anticipation, the posterior insula network was, therefore, overly engaged by ADP (less disengagement) compared with HC. Conversely, as previously mentioned, our results revealed diminished engagement of the SN in ADP during anticipation of risky trials. These findings suggest that the insula may be “malfunctioning” in different ways depending on the network. Understanding how each of these networks contributes to decisions and studying how these networks may be uniquely altered in disorders such as AD may give us a better understanding of the complex ways that the insula may influence behavior, specifically during risky decisions.

### Limitations and Future Directions

First, because of the time it takes to detect changes in a hemodynamic response, it is not possible to know exactly how networks engaged at anticipation versus decision execution are distinctly contributing to the decision-making process. Furthermore, the decision feedback was temporally contiguous with the decision to stop in successful trials or to press on cue in busted trials, confounding decision-making with feedback. Because decision-making is likely a fluid event with cognitive processes occurring simultaneously, it is difficult to break it into discrete stages. However, the ability of our study to identify differences in network engagement from the initiation of a trial (anticipation) to the termination (decision execution) indicates the importance of studying decisions as complex cognitive processes that evolve over time and, consequently, may heavily rely on effective network switching. Additionally, our findings demonstrate that the deviations in cognitive processing that occur in disorders such as AD may also change over the course of a cognitive task. Therefore, only observing network behavior at one point in the process may not give an accurate depiction of how the process is disrupted. For example, if we had only examined network engagement at trial anticipation the neural processing between ADP and HC would have looked deceivingly similar.

Second, because neither HC nor ADP demonstrated a high number of busted trials, our analysis was restricted to successful trials, which may have prevented us from gaining valuable information as to how network engagement may be further altered in failed or busted trials.

Lastly, due to our modest sample size and unbalanced group numbers, the findings should be replicated with a larger population and equal group sizes. Furthermore, many participants in our ADP sample also had a history of either cocaine and or cannabis dependence or abuse. Although there are many similarities across substance dependence, future work should study pure AD to examine if there are any significant differences in study results.

## Conclusion

In conclusion, our results indicate that although ADP are capable of recognizing risky conditions, they fail to appropriately update network engagement throughout the decision-making process, resulting in indiscriminate processing of risky and safe decisions. Additionally, our findings underscore the ability of a network approach to provide further insight to current knowledge of the functional response of brain regions, such as the insula. This approach provides a more comprehensive understanding of how and in what networks these regions may be impacted by disorders. Given that ADP did not behave differently in the task, altered network engagement may reflect an impaired ability to organize network switches to differentiate between the execution of safe and risky decisions. Consequently, ADP may avoid risky decisions and the burdensome recruitment of networks needed for the continual assessment of risk, instead opting for the safe and easy reward available in the task. Disorganized network switching and altered engagement may be related to a malfunctioning SN. Specifically, due to evidence of altered SN engagement in ADP, we argue that future work should examine whether widespread network engagement under risk is directly contingent on proper SN engagement and how this network could serve as a potential target for treatment of ADP. Importantly, processing a risky decision as if it were safe may result in negative consequences in real world situations. Indeed, our findings are consistent with the realities of substance dependence. Though many ADP report “knowing” a drink will lead to negative consequences, ADP often fail to resist the drink. This maladaptive behavior may result from a failure to properly engage the neural resources necessary to act according to the knowledge of the risk.

## Author Contributions

All authors have made a substantial contribution to the interpretation of data and to the intellectual content of the article. JB conceptualized the study and gathered the data. XZ performed the data analysis. KS and XZ wrote the manuscript with contributions from RM and JB.

## Conflict of Interest Statement

The authors declare that the research was conducted in the absence of any commercial or financial relationships that could be construed as a potential conflict of interest.
