# Supplementary material for: Alcohol Dependence and Altered Engagement of Brain Networks in Risky Decisions
Source: Front Hum Neurosci. 2016 Mar 31;10:142. doi: 10.3389/fnhum.2016.00142 (PMC4814760; doi:10.3389/fnhum.2016.00142)
Supplement: Supplementary file 1 [file Presentation_1.PDF]

Alcohol Dependence and Altered Engagement of Neural Networks in Risky Decisions

X. Zhu[1*]; K. Sundby[1*]; J. M. Bjork[2]; R. Momenan[1+]

[1]BEI, LCTS, NIAAA, National Institutes of Health, MD

[2]Department of Psychiatry, Virginia Commonwealth University, Richmond, VA

[*]These authors contributed equally to this manuscript.

Supplementary Materials

Supplementary Methods

Task design

The risk-taking task included two additional task conditions that were not discussed in our analysis, high-penalty (HP) and motor control (MC). In HP trials signified by a red screen, subjects were required to terminate reward accrual before an undisclosed time limit that could occur 4, 6, 8, or 10s after the first press. In HP, busts resulted in the subtraction from previous winnings of the amount that would have been earned for that trial had the subject not busted. In motor control trials, indicated by a white background, subjects pressed a button twice in response to two different cues for no incentive and no risk of penalty. Subjects were required to first press in response to the "$" cue to initiate the trial and later to the word "press", indicating the end of the trial.

Supplementary results

Network engagement during decision anticipation:

We compared network engagement between LP and HP to determine if the level of risk revealed any differences in network engagement. As summarized in Table S1, only the visual network demonstrated significantly distinct behavior between LP and HP, exhibiting higher engagement in the anticipation of HP for both HC and ADP ($p<0.0001$). We also compared network engagement between the anticipation of NP and our control condition, MC, which involved neither reward nor risk. When comparing network engagement during anticipation of NP and MC, we found that both HC and ADP revealed variations in engagement according to the trial condition. ADP and HC exhibited higher engagement of the Motor 1 network during the anticipation of NP trials. ADP also demonstrated higher recruitment of the BGTN and Motor 2 networks in anticipation of NP compared with MC trials. Alternatively, we found that the posterior insula network and ECN3 were more engaged during the anticipation of MC trials by ADP. Only HC increased engagement of the auditory network for MC trials. The results of these within group comparisons are summarized in Table S2.

Table S1. Network Engagement Distinguishing Trial Types at Anticipation LP vs. HP

		HC		ADP	
		HP vs. LP		HP vs. LP	
IC	Network	p-value	t	p-value	t
20	Visual Network	0.0003**	3.835	0.000**	8.825

A positive t-value indicates higher engagement in the first trial type listed in the comparison. *Networks with significantly different levels of engagement between ADP and HC. *p<0.05. **p<0.001.

Table S2. Network Engagement Distinguishing Trial Types at Anticipation NP vs. MC

			HC		ADP	
			NP vs. MC		NP vs. MC	
IC			corr-p	t	corr-p	t
4	Posterior Insula	Posterior Insula	0.073	-2.242	0.013*	-3.015
9	Motor1	precuneous	0.000**	4.431	0.000**	4.242
11	Auditory	STG,ITG	0.005*	-3.373	0.167	-1.335
12	ECN3	mPFC	0.413	-0.442	0.013*	-2.936
13	Motor 2	ACC, PreCG	0.153	1.505	0.028*	2.578
16	BGTN	thalaums, accumbens, caudate	0.083	2.065	0.044*	2.326

A positive t-value indicates higher engagement in the first trial type listed in the comparison. *Networks with significantly different levels of engagement between ADP and HC. *p<0.05. **p<0.001.

Network engagement during decision execution:

We examined whether HC and ADP engaged networks differently during the decision execution of LP and HP trials. As shown in Table S3, we only found significantly greater engagement of the visual network in HP trials in HC (p<0.001). We also examined differences in network engagement between NP and MC trials at decision execution. Both ADP and HC engaged the posterior insula network and the visual network more for MC than NP trials. In addition, ADP demonstrated higher engagement of ECN3, DMN, and auditory networks during the decision execution of MC compared with NP trials. In contrast, both ADP and HC engaged ECN1, RECN, and the Motor 1 network more for NP trials. Furthermore, only ADP revealed heightened

engagement of the SN and BGTN network during decision execution of NP trials. Similarly, only HC revealed increased engagement of the LECN at decision execution of NP compared with MC trials. The results of the within group comparisons at decision execution are summarized in Table S4.

Table S3. Network Engagement Distinguishing Trial Types at Decision Execution LP vs. HP

		HC		ADP	
		HP vs. LP		HP vs. LP	
IC	Network	p-value	t	p-value	t
20	Visual Network	0.0001**	3.891	0.0875	1.727

A positive t-value indicates higher engagement in the first trial type listed in the comparison. *Networks with significantly different levels of engagement between ADP and HC. *p<0.05. **p<0.001.

Table S4: Network Engagement Distinguishing Trial Types at Decision Execution NP vs. MC

			HC		ADP	
			NP vs. MC		NP vs. MC	
IC	Network	Structures	corr-p	t	corr-p	t
1	SN	Dorsal anterior insula, OFC, ACC	0.078	1.767	0.010*	2.803
4	Posterior Insula	Posterior Insula	0.000*	-4.356	0.010*	-3.086
6	LECN	IFG	0.006*	3.012	0.062	1.807
7	ECN1	FP, mPFC	0.017*	2.609	0.000*	4.433
9	Motor1	precuneous	0.000*	8.057	0.000*	6.857
10	RECN	IFG, OFC	0.000*	6.295	0.015*	2.561
11	Auditory	STG,ITG	0.131	-1.419	0.016*	-2.510
12	ECN3	mPFC	0.063	-2.025	0.014*	-2.639
14	DMN	PCC, mPFC	0.078	-1.872	0.027*	-2.243
16	BGTN	thalaums, accumbens, caudate	0.173	1.218	0.010*	2.824
20	Visual	visual cortex	0.005*	-3.083	0.010*	-2.987

A positive t-value indicates higher engagement in the first trial type listed in the comparison. *Networks with significantly different levels of engagement between ADP and HC. *p<0.05. **p<0.001.

Behavioral Data

As indicated in table S5, we found no significant differences between ADP and HC in the number of busted trials, time between first and second press, and money earned during the task.

Table S5: Task Behavior between ADP and HC

	ADP average	HC average	p-value	t-value
Average number of busts in LP	6.563	7.824	0.312	2.040
Money earned in task	32.522	33.256	0.771	2.069
Seconds between first and second presses in LP	12.674	13.401	0.631	2.0370

Networks identified with ICA

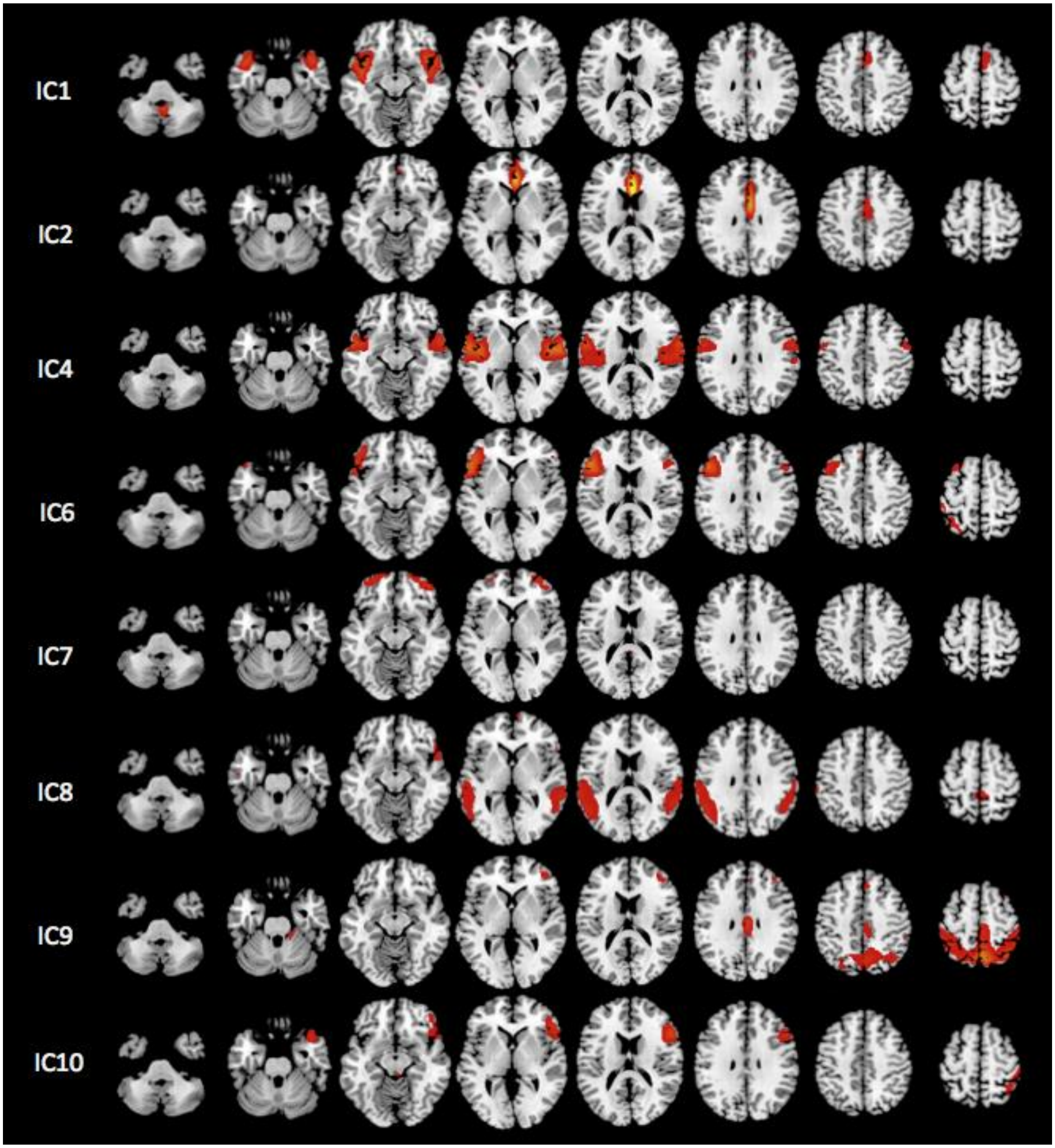

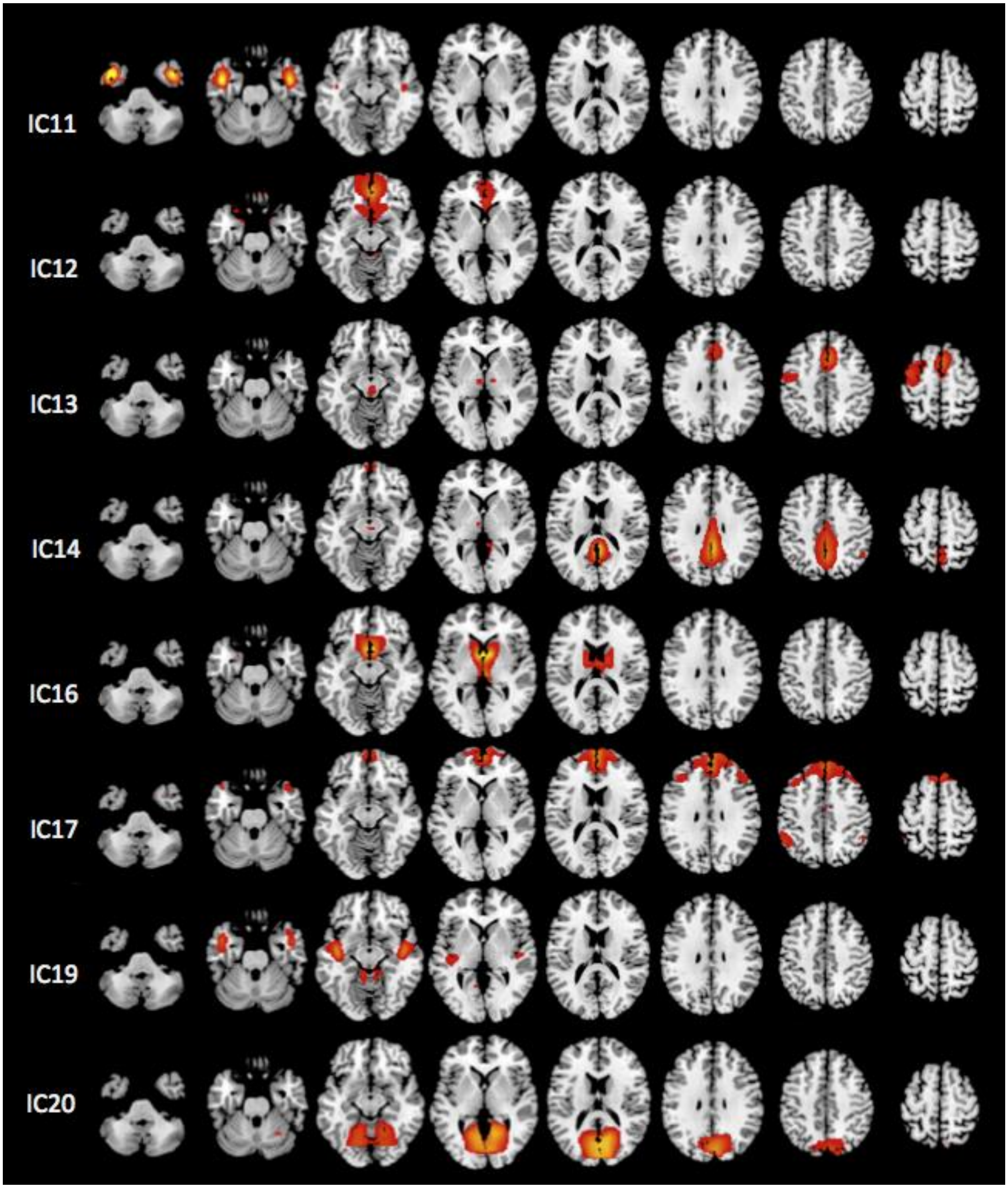

Figure S1: The ICA decomposition resulted in 20 spatial component maps. The IC3, IC5, IC15, and IC18 are brain stem, CSF or head motion that were identified as artifactual networks and removed from the study. IC1: Salience Network/dorsal anterior insula, IC2: ACC1, IC4: Posterior Insula, IC6: ECN1 including frontal pole and mPFC, IC7: ECN2 including angular gyrus and precentral gyrus, IC8: ECN3, IC9: Motor Network 1, IC10: RECN including

IFG and OFC, IC11: Auditory Network, IC12: ECN4 including mPFC, IC13: Motor Network2 including ACC and precentral gyrus, IC14: DMN including PCC and mPFC, IC16: BGTN including thalamus, caudate, nucleus accumbens, IC17: SFG, IC19: Ventral Anterior Insula, IC20: Visual Network
